# Gene expression changes as markers of early lapatinib response in a panel of breast cancer cell lines

**DOI:** 10.1186/1476-4598-11-41

**Published:** 2012-06-18

**Authors:** Fiona O’Neill, Stephen F Madden, Sinead T Aherne, Martin Clynes, John Crown, Padraig Doolan, Robert O’Connor

**Affiliations:** 1 Molecular Therapeutics for Cancer Ireland, National Institute for Cellular Biotechnology, Dublin City University, Glasnevin, Dublin 9, Ireland; 2 School of Nursing and Human Sciences, Dublin City University, Glasnevin, Dublin 9, Ireland

**Keywords:** Co-inertia analysis, Microarray, Lapatinib response, Breast cancer

## Abstract

**Background:**

Lapatinib, a tyrosine kinase inhibitor of HER2 and EGFR and is approved, in combination with capecitabine, for the treatment of trastuzumab-refractory metastatic breast cancer. In order to establish a possible gene expression response to lapatinib, a panel of breast cancer cell lines with varying sensitivity to lapatinib were analysed using a combination of microarray and qPCR profiling.

**Methods:**

Co-inertia analysis (CIA), a data integration technique, was used to identify transcription factors associated with the lapatinib response on a previously published dataset of 96 microarrays. RNA was extracted from BT474, SKBR3, EFM192A, HCC1954, MDAMB453 and MDAMB231 breast cancer cell lines displaying a range of lapatinib sensitivities and HER2 expression treated with 1 μM of lapatinib for 12 hours and quantified using Taqman RT-PCR. A fold change ≥ ± 2 was considered significant.

**Results:**

A list of 421 differentially-expressed genes and 8 transcription factors (TFs) whose potential regulatory impact was inferred *in silico*, were identified as associated with lapatinib response. From this group, a panel of 27 genes (including the 8 TFs) were selected for qPCR validation. 5 genes were determined to be significantly differentially expressed following the 12 hr treatment of 1 μM lapatinib across all six cell lines. Furthermore, the expression of 4 of these genes (RB1CC1, FOXO3A, NR3C1 and ERBB3) was directly correlated with the degree of sensitivity of the cell line to lapatinib and their expression was observed to “switch” from up-regulated to down-regulated when the cell lines were arranged in a lapatinib-sensitive to insensitive order. These included the novel lapatinib response-associated genes RB1CC1 and NR3C1. Additionally, Cyclin D1 (CCND1), a common regulator of the other four proteins, was also demonstrated to observe a proportional response to lapatinib exposure.

**Conclusions:**

A panel of 5 genes were determined to be differentially expressed in response to lapatinib at the 12 hour time point examined. The expression of these 5 genes correlated directly with lapatinib sensitivity. We propose that the gene expression profile may represent both an early measure of the likelihood of sensitivity and the level of response to lapatinib and may therefore have application in early response detection.

## Introduction

Breast cancer is the second most common malignancy in the world to date [1]. Classification of this cancer is based on a number of aspects such as tumour progression and pathology, estrogen receptor status and Human Epidermal growth factor Receptor 2 (HER2) status. All of these clinical parameters dictate the most suitable patient treatment.

HER2-positive breast cancer, in which the HER2 receptor is either overexpressed or amplified, is represented in approximately 20–30% of human breast cancers [2] and has been associated with poorer prognosis [3,4]. As with many cancers, there are a number of treatment options available to treat HER2 positive breast cancer. Radiation, surgery and chemotherapy have long been the standard for treatment. However, in recent years a more targeted approach has been taken in regards to treatment. Current targeted therapies available for this breast cancer subtype include the monoclonal antibody trastuzumab and the dual tyrosine kinase inhibitor lapatinib. The adverse effects associated with these types of therapies are less severe than those of traditional chemotherapies as they target cancer cells more specifically [5]. Tyrosine kinases are a group of enzymes that play a critical role in the signalling cascades of the cell. The tyrosine kinase functionality of these enzymes is typically coupled to and moderated by ligand binding (receptor) components and receptor-coupled tyrosine kinases are involved in the phosphorylation of tyrosine receptors in targeted proteins. Many important receptor-coupled tyrosine kinases are located in the cell membrane and proteins are activated by the binding of ligands to their extracellular domain. HER2 and EGFR (epidermal growth factor receptor) are two such examples of growth factor receptors which can homodimerise or dimerise with other members of the Human Epidermal Growth Factor Receptor family, which in turn activates their tyrosine kinase moiety. The activated tyrosine kinases have critical roles in cell signalling processes such as cell proliferation and growth [6,7]. Tyrosine kinase inhibitors (TKIs) prevent the activation of these tyrosine kinases thus inhibiting the activation of the pathways that would promote tumour cell growth and proliferation.

In this study, we focused on lapatinib, a dual kinase inhibitor developed by GlaxoSmithKline, which targets both HER2 and EGFR [8]. By binding to both HER2 and EGFR receptors, lapatinib prevents activation of important pro-cancer pathways such as Erk/MAPK (extracellular-signal-regulated kinase/mitogen-activated protein kinase) and PI3K (Phosphatidylinositol 3-kinases) which have vital roles in cell proliferation and survival [8,9]. Lapatinib is currently approved for treatment of metastatic breast cancer in combination with capecitabine [10]. It has also been used in combination with trastuzumab in patients suffering from advanced HER2 positive breast cancer [11].

Despite the wide application of HER2 testing in breast cancer, a significant proportion of HER2-positive patients do not respond to HER2-targeted therapy. In recent studies performed using lapatinib as a monotherapy, in combination with capecitabine and also with trastuzumab, clinical benefit response rates were found to range from 12.4% with lapatinib alone, 22% in combination with capecitabine and 24.7% in combination with trastuzumab [10,12,13].We have therefore sought to use cellular models to examine and identify the gene expression changes which might be characteristic of response to treatment with lapatinib.

In this paper, we used a multivariate statistical technique called co-inertia analysis (CIA) to link transcription factor binding site (TFBS) target predictions and gene expression data to identify transcription factors (TFs) associated with the cellular response to lapatinib [14,15]. This is the first time this data integration technique has been applied to a data set of breast cancer cells responding to drug treatment. The TFBS target predictions have been previously published [14]. In total this analysis contained TFBS information for 1236 known and predicted TFBSs across the conserved proximal promoters for ~17,000 genes. The gene expression dataset has been described previously [16] and incorporates time series data post treatment with high and low dose lapatinib in BT474 and SKBR3 cell lines.

From the original analysis [16] of this time series data, a number of gene expression changes were identified following treatment with lapatinib. These included a number of differentially expressed genes associated with the AKT pathway. This pathway is highly associated with cell proliferation, apoptosis and cell migration. The differentially regulated genes included, FOXO3A, CDKN1B, CCND1, AKT1 and E2F3. Of these genes, the authors focused on the expression of FOXO3A and some of its associated targets and regulators such as CDKN1B and CCND1 [16].

CIA is used to combine two linked datasets (two sets of measurements on the same objects) and perform two simultaneous non-symmetric correspondence analyses (NSC) and identify the axes that are maximally co-variant [15,17]. The use of an ordination method such as NSC or principle components analysis (PCA) allows us to summarise the data in a low dimensional space. In this case, the two linked datasets are normalised gene expression data from the lapatinib-treated cell lines and TFBS information for the same genes. We have previously used this method to compare gene expression data with miRNA target information [18] and proteomics data [19]. This is the first time that this approach has been used to analyse data derived from breast cancer cells responding to targeted therapy treatment.

CIA allows us to identify commonality between the expression of the genes and the TFs that are predicted to target these genes. It can be performed both unsupervised and supervised. The unsupervised step allows for data exploration and the identification of interesting trends or splits in the data and the supervised step allows us to identify which TFs are responsible for these splits. The supervised step incorporates the between group analysis (BGA) classification method [20,21] which is used in combination with the ordination method, forcing the ordination to be carried out on groups of samples rather than individual samples. First, a normal NSC is performed; BGA then finds the linear combination of the NSC axes that maximizes between-group variance and minimizes within-group variance, for specified groups. The output from this analysis is a ranked list of TFs predicted to be associated with the cellular response to lapatinib.

Using this approach, we were able to identify 8 TFs associated with the cellular response to lapatinib. This information was then used to generate a shortlist of 19 genes based on; the magnitude of their response to lapatinib, whether they were predicted targets of the 8 TFs and the involvement of the gene in important oncogenic processes. Genes were manually selected on the basis of meeting two or more of these criteria and as representatives to validate the typically less quantitative array data analyses. This cohort of 27 genes was examined using Taqman RT-PCR in a panel of 6 cell lines that had varying sensitivities to lapatinib. 5 genes were significantly differentially expressed across all 6 cell lines (RB1CC1, FOXO3A, NR3C1, ERBB3 and CCND1) and the expression of these 5 genes was directly correlated with the degree of sensitivity of each cell line to lapatinib.

## Materials and methods

### Gene expression data

The lapatinib-treated cell line dataset and experimental design has been described previously [16] and was obtained from the corresponding author in the form of raw data files (.cel files). The normalised data file can be downloaded from http://www.ebi.ac.uk/arrayexpress (accession number: E-MEXP-440). Gene expression values were called using the robust multichip average method [22] and data were quantile normalized using the Bioconductor package, affy. Affymetrix human genome HG-U133A arrays containing >22,000 probesets were used in this experiment. Briefly, the experimental design was as follows; four cell lines (BT474, SKBR3, T47D and MDAMB468) were analysed at 2, 6 and 12 hours post treatment with 0.1% DMSO (the control), 0.1 μM lapatinib and 1.0 μM lapatinib, with four replicates for each time point/treatment. In addition, 0.1% DMSO-treated cells were arrayed at 0 and 24 hours and 0.1 μM lapatinib treated cells were arrayed at 24 hours. Again these were arrayed in quadruplicate. In total, there were 48 arrays for each cell line. Our analysis focused on the two lapatinib sensitive cell lines, BT474 and SKBR3, comprising a total of 96 arrays (including controls).

Differential gene expression lists were generated using the ebayes function of the limma [23] package from Bioconductor. A fold change of ≥ 1.3 and an adjusted p-value of ≤ 0.05 were considered significant. The p-values are adjusted using the Benjamini and Hochberg method [24]. The choices of comparisons within the datasets were guided by the unsupervised CIA. In total there were 6 comparisons and these are summarised in Table 1. The final gene list was determined by consistent overlap between these 6 comparisons.

**Table 1:** A breakdown of the 6 comparisons for BT474 and SKBR3

**Comparison**	**Cell Line**	**Groups**	**Treatment**	**Time Point**	**Sample Number**
1	BT474	Group 1	1 μM lapatinib	6 hr & 12 hr	8
		Group 2	0.1 μM lapatinib	2 hr & 6 hr & 12 hr & 24 hr	16
			1 μM lapatinib	2 hr	4
			0.1% DMSO	0 hr & 2 hr & 6 hr & 12 hr & 24 hr	20
		**Total**			**48**
2	BT474	Group 1	1 μM lapatinib	6 hr & 12 hr	8
		Group 2	0.1 μM lapatinib	6 hr & 12 hr	8
		**Total**			**16**
3	SKBR3	Group 1	1 μM lapatinib	6 hr & 12 hr	8
			0.1 μM lapatinib	6 hr & 12 hr	8
		Group 2	0.1 μM lapatinib	2 hr & 24 hr	8
			1 μM lapatinib	2 hr	4
			0.1% DMSO	0 hr & 2 hr & 6 hr & 12 hr & 24 hr	20
		**Total**			**48**
4	SKBR3	Group 1	1 μM lapatinib	6 hr & 12 hr	8
			0.1 μM lapatinib	6 hr & 12 hr	8
		Group 2	0.1 μM lapatinib	2 hr	4
			1 μM lapatinib	2 hr	4
			0.1% DMSO	0 hr & 2 hr & 6 hr & 12 hr & 24 hr	20
		**Total**			**44**
5	SKBR3	Group 1	1 μM lapatinib	12 hr	4
			0.1 μM lapatinib	12 hr	4
		Group 2	0.1 μM lapatinib	2 hr & 6 hr & 24 hr	12
			1 μM lapatinib	2 hr & 6 hr	8
			0.1% DMSO	0 hr & 2 hr & 6 hr & 12 hr & 24 hr	20
		**Total**			**48**
6	SKBR3	Group 1	1 μM lapatinib	12 hr	4
			0.1 μM lapatinib	12 hr	4
		Group 2	0.1 μM lapatinib	2 hr & 6 hr	8
			1 μM lapatinib	2 hr & 6 hr	8
			0.1% DMSO	0 hr & 2 hr & 6 hr & 12 hr & 24 hr	20
		**Total**			**44**

The validity of choosing these six comparisons was confirmed by differentially expression analysis to show that early response in both BT474 and SKBR3 cells and low dose lapatinib in BT474 cells results in little or no lapatinib responsive genes. As above the Bioconductor package, Limma was used, and a fold change of ≥ 1.3 and an adjusted p-value of ≤ 0.05 were considered significant.

### Co-inertia analysis

CIA, a multivariate coupling technique, was used in an unsupervised manner to combine the two linked datasets; gene expression data from lapatinib-treated BT474 and SKBR3 cell lines and predicted TFBS information for the same genes. This initial step was used for data exploration and uses NSC. The analysis was then rerun in a supervised manner using BGA [14]. The output from this analysis is a ranked list of TFs predicted to be associated with the cellular response to lapatinib. The same 6 comparisons used to generate the differentially expressed gene list were used to generate 6 ranked lists of TFs. The final TF list was determined by overlap between these 6 ranked lists. All calculations were carried out using the MADE4 library [25] of the open source R package. MADE4 can be downloaded freely from the Bioconductor web site http://www.bioconductor.org. All the scripts and datasets used are available upon request from the authors.

### Transcription factor binding site information

The TFBS data has been previously published and contains information for 1236 known and predicted TFBSs across the conserved proximal promoters for ~17,000 genes at four different position specific scoring matrix (PSSM) thresholds, 0.7, 0.75, 0.8 and 0.85, giving 4 gene/TFBS frequency tables [14]. Using BGA with CIA, we were able to combine this information with gene expression data to gives 4 ranked lists of TFBS associated with a particular split of interest within the data; in this case, TFBS associated with the cellular response to lapatinib, for which we can infer the TFs linked with this response. The four lists were combined using the Rank Products method [26] which was initially developed for combining lists of differentially expressed genes. This gives one final list of ranked TFs.

### Statistical overrepresentation of TFBS

The TFs identified from the supervised CIA were validated using statistical overrepresentation of their predicted target genes within the differentially expressed gene list. A one-tailed fisher exact test was used as we are specifically interest in overrepresentation only [27,28]. The 421 consistently differentially expressed genes and the 8252 genes for which promoter information was available and were present on the U133A arrays, acted as the foreground and background for the fisher exact test respectively. The TFBS information is described in the previous section.

### Cell culture

SKBR3, HCC1954, EFM192A, MDAMB453 and MDAMB231 breast cancer cell lines were maintained in RPMI 1640 medium supplemented with 10% fetal bovine serum (PAA Labs, Austria). BT474 cells were maintained in Dulbeccos Modified Eagles medium (DMEM) supplemented with 10% fetal bovine serum, 2% L-Glutamine (Sigma, St Louis, MO, USA) and 1% Sodium Pyruvate (Sigma). All cell lines were kept at 37 °C in a 5% CO_2_/95% air humidified incubator.

### Lapatinib treatment and RNA extraction

Triplicate samples were grown to approximately 75% confluency. Treated samples were conditioned with 1 μM lapatinib for 12 hours. Control samples remained untreated. After the 12 hour incubation, the control and treated samples underwent RNA isolation using a Qiagen RNeasy mini Kit (Qiagen, Hilden, Germany) according to the manufacturer’s protocol and treated with Qiagen RNase-free DNase. cDNA template was then prepared from 2 μg of total RNA using an Applied Biosystems high capacity RNA to cDNA kit (Applied Biosystems, Foster City, CA, USA).

### Taqman RT PCR

TaqMan gene expression experiments were performed in 10 μl reactions in Taqman Array 96 well fast plates which had been pre-seeded with assays for the genes of interest. 40 ng of cDNA template and 5 μl of Taqman fast Universal Master Mix (2x), no AmpErase UNG (Applied Biosystems, Foster City, CA, USA) were dispensed into each well. The following thermal cycling specifications were performed on the ABI 7900 Fast Real-Time PCR system (Applied Biosystems, Foster City, CA, USA); 20 s at 95 °C and 40 cycles each for 3 s at 95 °C and 30 s at 60 °C. Expression values were calculated using the comparative threshold cycle (C_t_) method [29]. Glyceraldehyde-3-phosphate dehydrogenase (GAPDH) was selected as the endogenous control. The threshold cycle (C_t_) indicates the cycle number by which the amount of amplified target reaches a fixed threshold. The C_t_ data for GAPDH was used to create ΔC_t_ values [ΔC_t_ = C_t_ (target gene)-C_t_ (GAPDH)]. ΔΔC_t_ values were calculated by subtracting ΔC_t_ of the calibrator (untreated controls) from the ΔC_t_ value of each target. Relative quantification (RQ) values were calculated using the equation 2^-ΔΔCt^. Genes with a fold change ± 2 in the BT474 and SKBR3 cell lines were deemed to be differentially expressed.

### Proliferation assay in vitro

Cells were cultured in 96 well flat bottomed plates for 24 h before they were exposed to a range of concentrations of lapatinib for 6 days (0–20 μM for the insensitive cell lines and 0–1.5 μM for the sensitive cell lines). The % cell survival was then determined using an Acid Phosphatase assay. Media was removed from plates, the wells were washed twice with PBS and the cells were exposed to 10 mM PNP substrate in 0.1 M sodium acetate for approximately 1 hour. The reaction was stopped using 1 M NaOH and the plates were read at 405 nm and 620 nm on the plate reader (Synergy HT, Bio-Tek). The % cell survival was calculated as a percentage of non-treated controls.

## Results

### Unsupervised co-inertia analysis identifies prominent trends in the BT474 and SKBR3 cell lines

**Figure 1:**
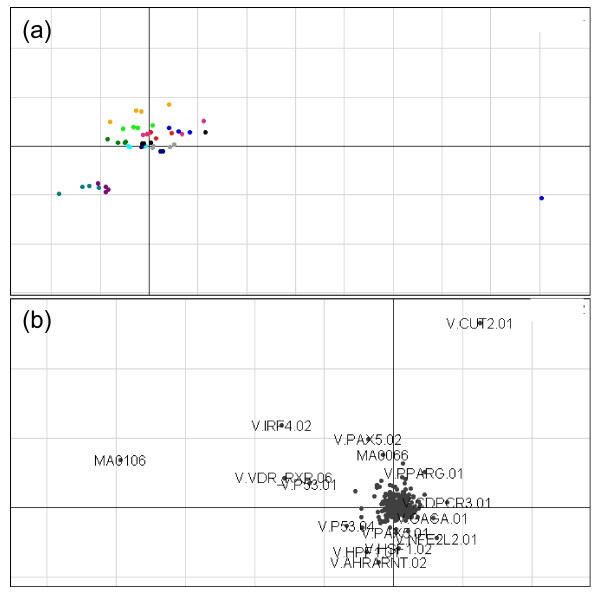
**Axes 1 (horizontal) and 3 (vertical) of the unsupervised CIA for BT474 cell line data**. A gene/TFBS frequency table produced with a PSSM threshold of 0.8 was used. **(a)** shows the projection of the cell line samples. The 0.1% DMSO treated samples (black 0 hr, red 2 hr, light blue 6 hr, light green 12 hr and orange 24 hr) and the 0.1 μM lapatinib treated samples (magenta 2 hr, dark blue 6 hr, cyan 12 hr and dark green 24 hr) are split from the 1 μM lapatinib treated samples (purple 6 hr and pale blue 12 hr). The exception being the four 1 μM lapatinib treated samples at 2 hours post treatment (grey). **(B)** Shows the projection of the TFBS motifs. Motifs that are in the same orientation (direction from the origin) as a group of samples are associated with those samples.

**Figure 2:**
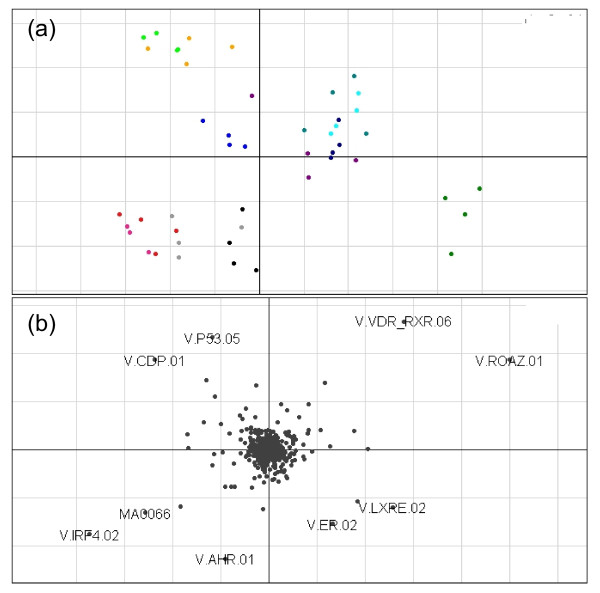
**Axes 1 (horizontal) and 2 (vertical) of the unsupervised CIA for SKBR3 cell line data**. A gene/TFBS frequency table produced with a PSSM threshold of 0.8 was used. Figure2**(a)** shows the projection of the cell line samples. The 0.1% DMSO treated samples (black 0 hr, red 2 hr, light blue 6 hr, light green 12 hr and orange 24 hr), are split from the 0.1 μM lapatinib treated samples (dark blue 6 hr, cyan 12 hr and dark green 24 hr) and the 1 μM lapatinib treated samples (purple 6 hr and pale blue 12 hr). The exception being the eight 0.1 μM lapatinib and 1 μM lapatinib treated samples at 2 hours post treatment coloured magenta and grey respectively. Figure,2**(b)** Shows the projection of the TFBS motifs. Motifs that are in the same orientation (direction from the origin) as a group of samples are associated with those samples.

For each cell line (BT474 and SKBR3) we used CIA to simultaneously analyse mRNA expression levels and TFBS information in the promoters of the same genes. Unsupervised CIA was applied to the 48 microarrays for each of the BT474 and SKBR3 cell lines and the associated gene/TFBS frequency tables to identify underlying trends in the data in each of the cell lines. The ultimate goal was to identify the TFs responsible for these trends and the differentially regulated genes they were predicted to target. The unsupervised CIA of the BT474 and SKBR3 cell lines are shown in Figures 1 and 2 respectively and are described in the following sections.

### Unsupervised co-inertia analysis of the BT474 cell line identifies a separation of 6- and 12-hour 1 μm lapatinib treatment samples

Axes one and three of the CIA for BT474 are plotted in Figure1a, for data exploration purposes. This allows us to estimate the response to lapatinib in the BT474 cell line. Axes one and three were chosen as they represent the dominant split within the data. The samples are labelled based on time and treatment. The samples at 6 hours and 12 hours post treatment with 1 μM lapatinib (purple 6 hr and pale blue 12 hr) clearly separated from those treated with 0.1% DMSO (black 0 hr, red 2 hr, light blue 6 hr, light green 12 hr and orange 24 hr), with 0.1 μM lapatinib (magenta 2 hr, dark blue 6 hr, cyan 12 hr and dark green 24 hr) and 2 hours post treatment with 1 μM lapatinib (grey), demonstrating a clear separation in the data between 1 μM lapatinib treated cells and the other samples. However, there was no difference between 0.1 μM lapatinib-treated and 0.1% DMSO-treated cells, suggesting that this is a dosage-dependent response in that a separation only occurred between the control samples and the high dose lapatinib samples, with the exception of one outlier on the far right of the plot. The lack of separation at 2 hours post treatment with 1 μM lapatinib suggests that the gene expression effects of the drug are not yet apparent at this time point. These observations guided our choice of comparisons for both the supervised CIA and the differential gene expression analysis which are summarised in Table 1.

Figure1b shows the motifs associated with this trend. The most extreme motifs along each axis are labelled and named. Those motifs furthest from the origin in the same orientation as the split of interest are most associated with that split. In this case V. AHRARNT.02 was the motif most associated with the separation of 1 μM lapatinib treated cells from the other samples and therefore is the motif most associated with the response to lapatinib. This is the motif for the agonist-activated heterodimer AHR/ARNT (Aryl hydrocarbon receptor/Arnt (hypoxia inducible factor 1 beta)) which directly associates with the estrogen receptors ER-alpha and ER-beta in ER-positive breast cancer, although its function in HER2-positive breast cancers is not well characterised [30].

### Unsupervised co-inertia analysis of the SKBR3 cell line identifies a separation of 6- and 12-hour 0.1 μM and 1 μm lapatinib treatment samples

Figure2a shows axes one and two of the CIA for SKBR3. The samples are labelled as before based on time and treatment. There was a clear split between the 0.1 μM (dark blue 6 hr and cyan 12 hr) and 1 μM (purple 6 hr and pale blue 12 hr) lapatinib-treated cells at 6 and 12 hours post treatment from the 0.1% DMSO treated controls (black 0 hr, red 2 hr, light blue 6 hr, light green 12 hr and orange 24 hr), with the exception of one outlier. As with the BT474 cell line there was no separation at 2 hours post treatment with 0.1 μM and 1 μM lapatinib coloured magenta and grey respectively, suggesting that the affects of the drug are not yet apparent at this time point in both cell lines. However, in this cell line the split occurred at both lapatinib dosages. Again, as with the BT474 data, these analyses were used to guide our comparisons for the supervised CIA and the expression analysis (Table 1).

The motifs associated with this split in the data are in the same orientation relative to the origin to our split of interest in Figure2b. These include the VDR/RXR heterodimer (V.VDR_RXR.06, vitamin D receptor/retinoid X receptor). This heterodimer has been previously associated with numerous cancers, including breast cancer [31].

### Validation of the 6 comparisons chosen for supervised CIA

The results from unsupervised CIA suggests that there was no difference between control and treated cells at both the high and low dose lapatinib at the 2 hour time point in both cell lines, and that there was no difference between treated and untreated BT474 cells at the 6 hr and 12 hr time point when low dose lapatinib was used. If this is the case there should be few differentially regulated genes at the early time point in both cell lines and at the low dose in the BT474 cell line. The results from these comparisons are shown in Additional file 1. On average there are ~60 differentially regulated genes in these comparisons compared to over ~2,500 differentially regulated genes when using the comparisons outlined in Table 1 (data not shown). This marked difference is a strong validation of our approach.

### Supervised CIA identifies 8 putative transcription factors associated with the response to lapatinib

In order to systematically identify the TFBSs specifically associated with the response to lapatinib in these cell lines (6 hr & 12 hr 1 μM lapatinib-treated samples vs. the other samples), we performed a supervised analysis of the data, combining CIA and BGA, as described. CIA was performed twice in the BT474 dataset and four times in the SKBR3 dataset (Table 1). This resulted in six ranked lists of TFBS associated with a response to lapatinib treatment (Additional file 2). The 6 transcription factor motifs (representing 8 individual transcription factors) which were consistently ranked highly across the six comparisons are displayed in Table 2. The individual ranking for each of the 6 comparisons are available in Additional file 2. From these motifs we can infer the 8 transcription factors which are driving the response to lapatinib in these cell lines.

**Table 2:** A ranked list of TFs associated with the response of BT474 and SKBR3 to lapatinib

**TF**	**Motif ID**	**Description**
RAR	V. RAR_RXR.02	Retinoic acid receptor
RXR	V.RAR_RXR.02	Retinoid X receptor
ARNT	V.AHRARNT.02	hypoxia inducible factor 1 beta
AHR	V.AHRARNT.02	Aryl hydrocarbon receptor
ZNF143	V.STAF.02	Zinc finger protein 143
PAX9	V.PAX9.01	Paired box gene 9
OLF1	V.OLF1.01	Olfactory neuron-specific factor
PAX3	V.PAX3.01	Paired box gene 3

### Differential gene expression analysis of the BT474 and SKBR3 cell lines identifies a list of 421 genes associated with response to lapatinib

The same six comparisons outlined in Table 1 were used to determine the genes which consistently respond to lapatinib treatment in both cell lines. In total, there were 421 distinct genes (274 probes upregulated and 244 probes downregulated) consistently dysregulated across the six comparisons. The full list of dysregulated genes, with associated fold-changes and p-values, is available in Additional file 3. A panel of 19 genes, in addition to the identified TFs, were selected for further analyses using qPCR based on varying combinations of the following criteria; (i) the magnitude of response to lapatinib, (ii) whether the selected genes were predicted targets of the 8 TFs, (iii) the involvement of the gene in important oncogenic processes (determined from functional annotation using the literature mining analysis software Pathway Studio Enterprise (Ariadne Genomics). Genes were manually selected on the basis of meeting two or more of these criteria and as representatives to validate the typically less quantitative array data analyses. These 19 genes are listed in Table 3 along with the TFs that are predicted to target them.

**Table 3:** Genes Selected for Taqman RT-PCR

**Gene Symbol**	**Gene Name**	**Key**	**Targeted by**
**CCND1**	**Cyclin D1**	^**1,3**^	**AHR/ARNT, PAX9, RAR/RXR**
**ERBB3**	**v-erb-b2 erythroblastic leukemia viral oncogene homolog 3**	^**2,3**^	**OLF-1**
**FOXO3**	**Forkhead box protein O3**	^**1,3**^	**RAR/RXR**
**NR3C1**	**nuclear receptor sub family 3 group C member 1**	^**2,3**^	**AHR/ARNT, PAX3**
**RB1CC1**	**RB1 inducible coiled coil protein 1**	^**3**^	**ZNF143**
ALDH3A2	aldehyde dehydrogenase 3 family member 2a	^2^+	
CDKN1B	cyclin dependent kindase inhibitor 1B	^2^+	
PIK3C3	phosphoinositide 3 kinase class 3	^2^+	
AKT1	v-akt murine thymoma viral oncogene homolog 1	^3^	RAR/RXR
BID	BH3 interacting domain	^2^	
E2F3	E2F transctription factor 3	^1,3^	AHR/ARNT, OLF-1, PAX9, ZNF143
eIF4E	eukaryotic translation initiation factor 4e	^3^	PAX3, RAR/RXR
FKBP4	Fk506 binding protein 4	^2,3^	ZNF143
MAPK9	mitogne-activated protein kinase 9	^2^	
PARP2	poly (ADP-ribose) polymerase 2	^2^	
PSMD13	proteasome 26 S subunit non-ATPase 13	^2^	
SLC29A1	solute carrier family 29 member 1	^2^	
TFPT	TCF3 (E2A) fusion partner	^2,3^	ZNF143
CBFA2T2	core-binding factor, runt domain, alpha subunit 2; translocated to, 2	^2^	

^1^ denotes a highly differentially regulated gene (±2 fold across all 6 comparisons) ^2^ denotes a gene that was known to be associated with cancer, ^3^ denotes a gene was predicted to be targeted by one or more of the transcription factors. Those in bold were found to be consistently dysregulated in response to lapatinib in all 6 of the cell lines. + denotes that the gene was found to be differentially expressed in BT474 and SKBR3 cell lines but not in the additional four cell lines also tested

### The predicted targets of the majority of the TFs identified by CIA are statistically overrepresented in the 421 genes associated with the response to lapatinib

In order to validate the results obtained by CIA we used a fisher exact test to determine if the predicted targets of the 8 TFs identified, were enriched in the 421 genes associated with the response to lapatinib. The results are shown in Additional file 4. The 8 TFs are represented by 6 motifs (Ahr/ARNT and RAR/RXR bind as heterodimers). Of these 6 motifs, 4 are significantly overrepresented in the promoters of the 421 lapatinib responsive genes (PAX9, p-value = 0.04, PAX3, p-value = 0.05, Ahr/ARNT, p-value = 0.002 and ZNF143, p-value = 0.0003), while two are not (OLF-1, p-value = 0.38, and RAR/RXR, p-value = 1). While none of these transcription factors are present in the 421 gene list their predicted targets are modulated in response to lapatinib for the majority of the TFs identified.

### Lapatinib toxicological analysis in a panel of cell lines using acid-phosphatase proliferation assay identifies a range of drug-responses in breast cancer

The IC50 values determined using the described methods were found to correlate with previous published data for 5 of the 6 cell lines (BT474, SKBR3, EFM192A, HCC1954 and MDAMB453) [2]. There are currently no publically available IC50 values for lapatinib response in MDAMB231 cells. The values determined were 0.036 ± 0.0151 μm for BT474, 0.080 ± 0.0173 μM for SKBR3, 0.193 ± 0.0665 μM for EFM192A, 0.4166 ± 0.18 μM for HCC1954, 6.08 ± 0.825 μM for MDAMB453 and 7.46 ± 0.102 μM for MDAMB231 (Table 4).

**Table 4:** IC50 values of selected cell lines

	Cell Line Name	IC50 ± SD (μM)
Lapatinib Sensitive Cell Lines	BT474	0.036 ± 0.0151
	SKBR3	0.080 ± 0.0173
	EFM 192A	0.193 ± 0.0665
	HCC1954	0.416 ± 0.180
Lapatinib Insensitive Cell Line	MDA MB 453	6.08 ± 0.825
Triple Negative Cell Line	MDA MB 231	7.46 ± 0.102

### Taqman PCR analysis confirms dysregulation of the 8 transcription factors following lapatinib exposure

The initial Taqman RT PCR analysis was carried out in lapatinib-treated BT474 and SKBR3 breast cancer cell lines. The drug concentration and treatment duration were also evaluated using the CIA. The combination of 1 μM lapatinib and 12 hours post treatment are the optimal conditions for treating the cells based on the separations seen in Figures 1 and 2. In addition, 1 μM of lapatinib is a clinically relevant concentration [8]. These two cell lines are highly sensitive to lapatinib with IC50 values of 0.036 μM ± 0.0151 μM and 0.080 μM ± 0.0173 μM respectively (Table 4) [2]. Four additional cell lines were also chosen based on their sensitivity to lapatinib (EFM192A, HCC1954, MDAMB453 and MDAMB231). Their IC50 values are shown in Table 4.

**Figure 3:**
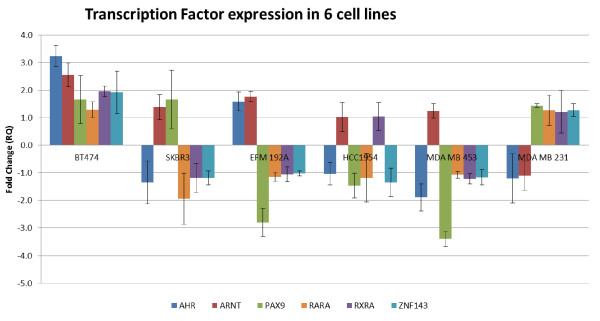
**Expression of Transcription Factors**. The transcription factor expression was calculated using ΔΔC_t_ values of the control and treated cell line samples. N = 3.

76 of the 8 transcription factors were found to be present following 1 μM 12 hr lapatinib treatment relative to untreated controls (Figure3). Although these genes were not identified from the differential gene expression analysis, they are clearly dysregulated in these cell lines, as predicted by CIA. 2 of the predicted transcription factors (PAX3 and OLF1) were not expressed (data not shown). While the expression of the transcription factors does not follow a set pattern, there are some distinct trends. For example, all the TFs were up-regulated in the most lapatinib-sensitive cell line (BT474) and nearly all down-regulated in the most lapatinib-insensitive cell line (MDA MB 453). In addition ARNT was up-regulated in all lines, apart from MDAMB231, the triple negative cell line.

### Taqman PCR analysis confirms a consistent dysregulation of 5 of the 19 additional genes selected for validation, following lapatinib exposure

A panel of 19 genes was selected from the list of 421 candidate genes as described (Table 3). As with the TFs, the 19 genes were first analysed for differential expression in BT474 and SKBR3 cells that had been treated with 1 μM lapatinib for 12 hours using untreated cells as a control. Of the 19 genes 5 were found to be differentially expressed with an RQ value of ≥ ±2 in both the BT474 and SKBR3 cell lines (RB1CC1, FOXO3, NR3C1, ERBB3 and CCND1) (RQ values for all genes are available in Additional file 5. Basal gene expression is available for these 5 genes in Additional file 6, Figure S2). Of the remaining 14 genes (AKT1, ALDH3A2, BID, CDKN1B E2F3, eIF4E, FKBP4, MAPK9, PARP2, PIK3C3, PSMD13, SLC29A1, TFPT and CBFA2T2), some were found to be differentially expressed, however, this alteration in expression did not occur in both of the cell lines and they were therefore excluded from further analysis.

For further validation expression of, CCND1, ERBB3, FOXO3, NR3C1 and RB1CC1, was analysed in two additional lapatinib-sensitive cell lines EFM192A and HCC1954. Both of these cell lines are HER2-positive and have varying sensitivities to lapatinib, with IC50 values of 0.193 μM ± 0.0665 μM and 0.4166 μM ± 0.18 μM, respectively. Two lapatinib-insensitive cell lines were also analysed, MDAMB453 and MDAMB231. MDAMB453 is a HER2-positive breast cancer cell line that is innately insensitive to lapatinib and MDAMB231 is a triple negative breast cancer cell line that has an IC50 value of 7.46 ± 0.102 μM

In the lapatinib sensitive cell lines (BT474, SKBR3, EFM192A and HCC1954), the 5 genes showed differential gene expression levels proportional to the degree of sensitivity of the cells to lapatinib and are highlighted in bold in Table 3.

**Figure 4:**
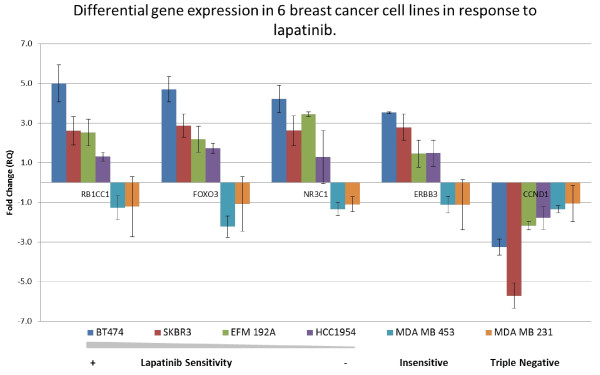
**Differential expression of 5 significant genes**. Analysis across the 6 cell lines showed that 5 genes were differentially regulated in response to lapatinib and the degree of dysregulation was proportional to the response to lapatinib. The cell lines are represented in order of sensitivity to lapatinib, with BT474 being the most sensitive and MDAMB231 being the least. N = 3.

Figure4 shows the expression profiles of RB1CC1, FOXO3A, NR3C1 and ERBB3 in the four lapatinib-sensitive cell lines and clearly demonstrates a correlation between the degree of sensitivity of the cell line to lapatinib and the magnitude of differential gene expression. BT474 was the most lapatinib-sensitive cell line and displayed the highest differential expression values for the four upregulated genes. As the cell lines became less sensitive to lapatinib, the magnitude of differential gene expression decreases. In the lapatinib-insensitive cell lines, MDAMB453 and MDAMB231, the expression of these genes “switched” from up-regulation following lapatinib exposure to down-regulation.

In the case of CCND1, the differential gene expression pattern followed a largely proportional response and not a “switching” response which was evident with the other genes. In the lapatinib-sensitive cell lines the gene was found to be strongly down-regulated following the 12 hr treatment. The magnitude of this down-regulation was reduced as the cells became more lapatinib-insensitive (Figure4).

## Discussion

In this paper, we describe the application of a method (CIA) for inferring the action of TFs by integrating the information provided by TFBS target prediction with mRNA gene expression data [14] to identify possible markers for early lapatinib response. This is the first time this approach has been used to analyse an array data set derived from breast cancer cells treated with a targeted therapeutic. This multivariate statistical technique was applied to gene expression data incorporating time series data post treatment with high and low dose lapatinib in lapatinib-sensitive, HER2-positive cell lines (48 microarrays on both BT474 and SKBR3 cell lines). This method was initially used for data exploration to determine the gene expression response to lapatinib. This response appears to require a high dose of lapatinib in BT474 cells (1 μM lapatinib) and require low to high dose in SKBR3 cells (0.1 μM or 1 μM lapatinib). Differential gene expression analysis at early times or lose dose lapatinib confirmed this, as we were unable to identify a substantial gene list at low dose lapatinib in BT474 cells and at the 2 hr time point in either cell line, providing a strong validation of our approach. Once the lapatinib response was determined, CIA was used in a supervised manner to identify 8 TFs associated with response to lapatinib. It is important to note that none of these TFs were associated with the lapatinib response through standard differential expression analysis and their prioritisation here was only achieved via the novel use of the CIA method in this breast cancer dataset. Statistical overrepresentation of these TFs in the promoters of the 421 differentially regulated genes was used to further confirm of the validity of the approach we used here. 4 of the 6 motifs (representing the 8TFs) were statistically overrepresented in the lapatinib responsive gene list (PAX9, PAX3, Ahr/ARNT and ZNF143). While OLF-1 and RAR/RXR expression levels were not statistically significant within this gene list, it is not unexpected, as CIA is not restricted to a specific gene list but rather uses the entire microarray data as input. CIA is therefore not limited by arbitrary cut-offs which may exclude important TFs of interest. Overall the target genes of the TFs identified by CIA show higher than expected modulation by lapatinib, even though the TFs themselves are not differentially regulated.

These 8 TFs and an additional 19 putative markers were then validated using qPCR in a panel of breast cancer cell lines following treatment with 1 μM lapatinib for 12hours. The results suggest that the 5 genes RB1CC1, NR3C1, FOXO3A, ERBB3 and CCND1, which had been found to be differentially regulated in response to lapatinib treatment could be utilised as potential markers for early lapatinib response as their expression correlates with the sensitivity of the cell lines to lapatinib.

The expression of 6 TFs, AHR, ARNT, RXR, RAR, PAX9 and ZNF143 were found to be altered across all the cell lines in response to lapatinib treatment. These TFs are putative regulators of the cellular response to lapatinib and are predicted to target a number of the significantly differentially regulated genes. The expression of these TFs does not follow a set pattern but do follow some distinct trends as mentioned above, however, the regulation of gene expression by TFs is difficult to discern directly from the expression pattern of the TF genes themselves. All of these TFs have been previously demonstrated to play important roles in cancer, although their function in HER2-positive breast cancer is unclear. The AHR/ARNT heterodimer has been implicated as having importance in ER positive breast cancer and has been shown to directly associate with estrogen receptors ER-alpha and ER-beta [30,32,33]. Retinoids targeting the RXR/RAR heterodimer have marked affects on cellular processes such as proliferation and apoptosis and this has been shown both *in vivo* and in vitro in breast cancer models [34]. The RARA receptor has also been recently identified as being co-amplified with HER2 in some breast cancers [35]. While being known oncogenes, PAX9 and ZNF143 have not been extensively studied in breast cancer [36,37] and none of these TFs have previously been implicated in the response to lapatinib.

From the panel of 5 genes, 4 were upregulated in response to lapatinib, RB1CC1, NR3C1, FOXO3A and ERBB3. The expression of these genes correlated with the sensitivity of each cell line to lapatinib. The results show that the more sensitive that the cell line is to lapatinib, which was determined using proliferation assays, the greater the magnitude of up-regulation of the 4 genes. The genes then “switch” to down-regulation in the remaining two lapatinib insensitive cell lines (MDAMB453 and MDAMB231). In the case of CCND1, this switching phenomenon is not evident; rather the expression of CCND1 becomes less down-regulated as the level of lapatinib sensitivity decreases.

All 5 of the genes have been previously demonstrated to have importance in cancer. RB1 inducible coiled-coil 1 (RB1CC1) expression has been shown to be associated with long term survival of breast cancer patients and has been found to have a role in the inhibition G1-S progression and proliferation in breast cancer cell lines [38,39]. NR3C1, a glucocorticoid receptor, has been associated with poor response to treatment in multiple myeloma samples [40]. Up-regulation of ERBB3 (HER3) has been connected with invasive breast carcinomas and also drug resistance in some HER2-overexpressing cancers [41].

FOXO3A and CCND1 have been demonstrated to be important in both breast cancer and the lapatinib response [16,42]. FOXO3A and CCND1 were both shown by [16] to be differently expressed following treatment with lapatinib. This group reported up-regulation of FOXO3A in both BT474 and SKBR3 and also a down-regulation of CCND1 in the same cell lines. These results are consistent with the results obtained by our study. It should be noted that CDKN1B was also differentially expressed in response to lapatinib in our study, [16] although its dysregulation did not correlate with lapatinib sensitivity (Additional file 6 figure S1). The authors identified that these three genes all played roles in the regulation of the AKT pathway, both positive and negative. They noted that the down regulation of CCND1 and that the upregulation of CDKN1B in response to lapatinib could be as a result of a FOXO3A-dependent mechanism, which promotes lapatinib-induced apoptosis. However, they did not examine the expression of these genes in other lapatinib sensitive cells lines nor did they observe that the expression of these genes correlated with the sensitivity of the cell lines to lapatinib. They also observed additional changes in response to genes associated with a number of cellular processes such as glycolysis and cell cycle regulation.

Interestingly, CCND1 links all of these genes together both at the TF level (it is predicted to be targeted by AHR/ARNT, RXR/RAR and PAX9) and at the gene level via several interactions. CCND1 was downregulated in response to lapatinib in our panel of cell lines which is consistent with previous studies [43-45] and which may also be related to its known interactions with our other genes of interest. FOXO3A has been shown to down-regulate CCND1 during cell cycle inhibition [46], while Erbb3 receptors are thought to be required to reduce transcription of E2F mediated transcription factors, which include CCND1 [47]. NR3C1 has been noted to inhibit CCND1 activation, through the TCF/ß-catenin complex [48,49] and RB1CC1 has been found to decrease the expression of CCND1 by promoting its degradation [39]. Also, AHR/ARNT has been shown to regulate cell cycle progression via a functional interaction with CDK4/CCND1 [32] and retinoids (RXR/RAR receptor ligands) are known to inhibit CCND1 expression [50].

Of the 5 lapatinib-responsive genes, FOXO3A and CCND1 were previously described in lapatinib-treated BT474 and SKBR3 cell lines by the group that generated the original microarray dataset [16]. However, the inclusion of the additional 4 cell lines allowed us to examine the 5 differentially expressed genes in the context of cell lines with varying sensitivities to lapatinib. The upregulation of RB1CC1 and NR3C1 in response to lapatinib has not been previously observed, while only limited work has been performed on ERBB3, FOXO3A and CCND1 in this setting. While the analysis described in this work is of a descriptive nature, a number of these genes including, FOXO3a (Mickey C.-T Hu *et al.)* and ERBB3 (Liu, B *et al.*) have been successfully functionally validated as being important in breast cancer response [51,52] .

The methods we have employed represent an attractive approach to dissection of the underlying gene expression changes associated with the response of cellular models of breast cancer (with differing inherent sensitivity) to lapatinib treatment. Our experimental design generated a list of gene changes that directly correlate with response to lapatinib in breast cancer. Since the list is highly enriched for genes likely to be of importance in lapatinib response, our findings therefore represent interesting candidates as biomarkers of response or functional targets for therapeutic intervention to improve response/overcome resistance.

## Conclusions

In summary, we used CIA to identify a number of genes and TFs associated with the cellular response to lapatinib. This is the first time that this technique has been applied to a dataset derived from drug-treated breast cancer cells. This panel included both known and novel markers of the lapatinib response and represents an ideal cohort of markers both for the response to lapatinib and the cellular sensitivity to lapatinib. The expression of 5 of these genes correlated directly with lapatinib sensitivity. We identified known lapatinib response genes such as FOXO3A, CDKN1B and CCND1, as well as novel responders to lapatinib, RB1CC1 and NR3C1. In addition, we have identified putative candidate regulators of this lapatinib response, none of which have been previously studied in lapatinib-treated cells. Since our methods highly enrich for genes likely to be of importance in the drug response, they represent a novel route to identification of putative response biomarkers or targets for therapeutic intervention to increase treatment efficacy.

## Abbreviations

HER2, Human epidermal growth factor Receptor 2; EGFR, Epidermal growth factor receptor; Erk/MAPK, Extracellular-signal-regulated kinase/mitogen-activated protein kinase; PI3K, Phosphatidylinositol 3-kinases; CIA, Co-inertia analysis; TFBS, Transcription factor binding site; TF, Transcription factors; NSC, Non-symmetric correspondance analyses; PCA, Principal components analysis; BGA, Between group analysis; PSSM, Position specific scoring matrix; GAPDH, Glyceraldehyde-3-phosphate dehydrogenase; PNP, P-nitrophenol phosphate; RQ, Relative quantification; VDR/RXR, Vitamin D receptor/retinoid X receptor; RB1CC1, RB1 inducible coiled-coil 1; RAR, Retinoic acid receptor; RXR, Retinoid X receptor; ARNT, Hypoxia inducible factor 1 beta; AHR, Aryl hydrocarbon receptor; ZNF143, Zinc finger protein 143; PAX9, Paired box gene 9; OLF1, Olfactory neuron-specific factor; PAX3, Paired box gene 3; AKT1, V-akt murine thymoma viral oncogene homolog 1; FOXO3, Forkhead box protein O3; CCND1, Cyclin D1; E2F3, E2F transcription factor 3; eIF4E, Eukaryotic translation initiation factor 4e; RB1CC1, Retinoblastoma 1 inducible coiled coil protein 1; ERBB3, V-erb-b2 erythroblastic leukemia viral oncogene homolog 3; MAPK9, Mitogen-activated protein kinase 9; FKBP4, Fk506 binding protein 4; TFPT, TCF3 (E2A) fusion partner; NR3C1, Nuclear receptor sub family 3 group C member 1; ALDH3A2, Aldehyde dehydrogenase 3 family member 2a; PIK3C3, Phosphoinositide 3 kinase class 3; BID, BH3 interacting domain; PARP2, Poly (ADP-ribose) polymerase 2; PSMD13, Proteasome 26 S subunit non-ATPase 13; SLC29A1, Solute carrier family 29 member 1; CDKN1B, Cyclin dependent kinase inhibitor 1B.

## Competing interests

The authors declare that they have no competing interests.

## Authors’ contributions

FON and SFM contributed equally to this work. SFM performed all of the bioinformatic/statistical analysis. FON treated the cells with lapatinib extracted the RNA and performed Taqman RT PCR and the proliferation assay. STA participated in the study design, RNA extraction and TaqMan RT PCR and analysis and interpretation of the results. FON, SFM, STA, JC, MC ROC and PD contributed to the result interpretation and manuscript preparation. ROC and PD equally conceived the study, participated in its design, coordination and interpretation of the results and finalized the manuscript. All authors read and approved the final manuscript.

## Supplementary Material

Additional File 1 Lapatinib modulated genes responding early or at low dosage. Click here for file

Additional File 2 A ranked list of TFBS associated with a response to lapatinib treatment for each of the 6 comparisons in 0 Table 1. Highlighted in bold are those TFBS consistent across the 6 comparisons. Click here for file

Additional File 3 A full list of the differentially regulated genes and the TFs that are predicted to target them. This file also contains the fold change and the adjusted p-value for each of the six comparisons. Click here for file

Additional File 4 Statistical Overrepresentation of the TFs identified by CIA. Click here for file

Additional File 5 RQ values for all genes tested, including the TFs. Click here for file

Additional File 6 **Figure S1.** Expression of PIK3C3, ALDH3A2 and CDKN1B across the six cell lines. **Figure S2**. Basal gene expression (ΔC_t_) of RB1CC1, FOXO3A, NR3C1, ERBB3 and CCND1 across the six cell lines. Click here for file

## References

[B1] Ferlay J Shin H Bray F Forman D Mathers C Parkin DM Estimates of worldwide burden of cancer in 2008: GLOBOCAN 2008 Int J Cancer 2010 127 2893 2917 10.1002/ijc.25516 21351269

[B2] O'Brien NA Browne BC Chow L Wang Y Ginther C Arboleda J Duffy MJ Crown J O'Donovan N Slamon DJ Activated phosphoinositide 3-kinase/AKT signaling confers resistance to trastuzumab but not lapatinib Mol Cancer Ther 2010 9 1489 1502 10.1158/1535-7163.MCT-09-1171 20501798

[B3] Slamon DJ Clark GM Wong SG Levin WJ Ullrich A McGuire WL Human breast cancer: correlation of relapse and survival with amplification of the HER-2/neu oncogene Science 1987 235 177 182 10.1126/science.3798106 3798106

[B4] Ross JS Fletcher JA The HER-2/neu oncogene in breast cancer: prognostic factor, predictive factor, and target for therapy Stem Cells 1998 16 413 428 10.1002/stem.160413 9831867

[B5] Sawyers C Targeted cancer therapy Nature 2004 432 294 297 10.1038/nature03095 15549090

[B6] Paul MK Mukhopadhyay AK Tyrosine kinase - Role and significance in Cancer Int J Med Sci 2004 1 101 115 15912202 10.7150/ijms.1.101 PMC1074718

[B7] Arora A Scholar EM Role of Tyrosine Kinase Inhibitors in Cancer Therapy J Pharmacol Exp Ther 2005 315 971 979 10.1124/jpet.105.084145 16002463

[B8] Burris HA Hurwitz HI Dees EC Dowlati A Blackwell KL O'Neil B Marcom PK Ellis MJ Overmoyer B Jones SF Harris JL Smith DA Koch KM Stead A Mangum S Spector NL Phase I safety, pharmacokinetics, and clinical activity study of lapatinib (GW572016), a reversible dual inhibitor of epidermal growth factor receptor tyrosine kinases, in heavily pretreated patients with metastatic carcinomas J Clin Oncol 2005 23 5305 5313 10.1200/JCO.2005.16.584 15955900

[B9] Rusnak DW Lackey K Affleck K Wood ER Alligood KJ Rhodes N Keith BR Murray DM Knight WB Mullin RJ Gilmer TM The effects of the novel, reversible epidermal growth factor receptor/ErbB-2 tyrosine kinase inhibitor, GW2016, on the growth of human normal and tumor-derived cell lines in vitro and in vivo Mol Cancer Ther 2001 1 85 94 12467226

[B10] Geyer CE Forster J Lindquist D Chan S Romieu CG Pienkowski T Jagiello-Gruszfeld A Crown J Chan A Kaufman B Skarlos D Campone M Davidson N Berger M Oliva C Rubin SD Stein S Cameron D Lapatinib plus capecitabine for HER2-positive advanced breast cancer N Engl J Med 2006 355 2733 2743 10.1056/NEJMoa064320 17192538

[B11] Konecny GE Pegram MD Venkatesan N Finn R Yang G Rahmeh M Untch M Rusnak DW Spehar G Mullin RJ Keith BR Gilmer TM Berger M Podratz KC Slamon DJ Activity of the dual kinase inhibitor lapatinib (GW572016) against HER-2-overexpressing and trastuzumab-treated breast cancer cells Cancer Res 2006 66 1630 1639 10.1158/0008-5472.CAN-05-1182 16452222

[B12] Gomez HL Doval DC Chavez MA Ang PC Aziz Z Nag S Ng C Franco SX Chow LWC Arbushites MC Casey MA Berger MS Stein SH Sledge GW Efficacy and safety of lapatinib as first-line therapy for ErbB2-amplified locally advanced or metastatic breast cancer J Clin Oncol 2008 26 2999 3005 10.1200/JCO.2007.14.0590 18458039

[B13] Blackwell KL Burstein HJ Storniolo AM Rugo H Sledge G Koehler M Ellis C Casey M Vukelja S Bischoff J Baselga J O'Shaughnessy J Randomized study of Lapatinib alone or in combination with trastuzumab in women with ErbB2-positive, trastuzumab-refractory metastatic breast cancer J Clin Oncol 2010 28 1124 1130 10.1200/JCO.2008.21.4437 20124187

[B14] Jeffery IB Madden SF McGettigan PA Perriere G Culhane AC Higgins DG Integrating transcription factor binding site information with gene expression datasets Bioinformatics 2007 23 298 305 10.1093/bioinformatics/btl597 17127681

[B15] Dolédec S Chessel D Co-inertia analysis: an alternative method for studying species - environment relationships Freshw Biol 1994 31 294 277

[B16] Hegde PS Rusnak D Bertiaux M Alligood K Strum J Gagnon R Gilmer TM Delineation of molecular mechanisms of sensitivity to lapatinib in breast cancer cell lines using global gene expression profiles Mol Cancer Ther 2007 6 1629 1640 10.1158/1535-7163.MCT-05-0399 17513611

[B17] Dray S Chessel D Thioulouse J Co-inertia analysis and the linking of ecological data tables Ecology 2003 84 3078 3089 10.1890/03-0178

[B18] Madden S Carpenter S Jeffery I Bjorkbacka H Fitzgerald K O'Neill L Higgins D Detecting microRNA activity from gene expression data BMC Bioinforma 2010 11 257 10.1186/1471-2105-11-257 PMC2885376 20482775

[B19] Fagan A Culhane AC Higgins DG A multivariate analysis approach to the integration of proteomic and gene expression data Proteomics 2007 7 2162 2171 10.1002/pmic.200600898 17549791

[B20] Dolédec S Chessel D Rhythmes saisonniers et composantes stationelles en milieu aquatique I—Description d'un plan d'observations complet par projection de variables Acta Oecologica Oecologica Generalis 1987 8 403 426

[B21] Culhane AC Perrière G Considine EC Cotter TG Higgins DG Between-group analysis of microarray data Bioinformatics 2002 18 1600 1608 10.1093/bioinformatics/18.12.1600 12490444

[B22] Irizarry RA Hobbs B Collin F Beazer-Barclay YD Antonellis KJ Scherf U Speed TP Exploration, normalization, and summaries of high density oligonucleotide array probe level data Biostatistics 2003 4 249 264 10.1093/biostatistics/4.2.249 12925520

[B23] Smyth GK Linear models and empirical bayes methods for assessing differential expression in microarray experiments Stat Appl Genet Mol Biol 2004 3 Article3 16646809 10.2202/1544-6115.1027

[B24] Benjamini Y Hochberg Y Controlling the False Discovery Rate: A Practical and Powerful Approach to Multiple Testing J Royal Statistical Soc Series B (Methodological) 1995 57 289 300

[B25] Culhane AC Thioulouse J Perrière G Higgins DG MADE4: an R package for multivariate analysis of gene expression data Bioinformatics 2005 21 2789 2790 10.1093/bioinformatics/bti394 15797915

[B26] Breitling R Armengaud P Amtmann A Herzyk P Rank products: a simple, yet powerful, new method to detect differentially regulated genes in replicated microarray experiments FEBS Lett 2004 573 83 92 10.1016/j.febslet.2004.07.055 15327980

[B27] Ho Sui SJ Fulton DL Arenillas DJ Kwon AT Wasserman WW oPOSSUM: integrated tools for analysis of regulatory motif over-representation Nucleic Acids Res 2007 35 W245 W252 10.1093/nar/gkm427 17576675 PMC1933229

[B28] Leonard MO Howell K Madden SF Costello CM Higgins DG Taylor CT McLoughlin P Hypoxia selectively activates the CREB family of transcription factors in the in vivo lung Am J Respir Crit Care Med 2008 178 977 983 10.1164/rccm.200712-1890OC 18689465 PMC2643223

[B29] Livak KJ Schmittgen TD Analysis of relative gene expression data using real-time quantitative PCR and the 2(−Delta Delta C(T)) Method Methods 2001 25 402 408 10.1006/meth.2001.1262 11846609

[B30] Rüegg J Swedenborg E Wahlström D Escande A Balaguer P Pettersson K Pongratz I The transcription factor aryl hydrocarbon receptor nuclear translocator functions as an estrogen receptor beta-selective coactivator, and its recruitment to alternative pathways mediates antiestrogenic effects of dioxin Mol Endocrinol 2008 22 304 316 17991765 10.1210/me.2007-0128 PMC5419643

[B31] Conde I Paniagua R Fraile B Ruiz A Arenas MI Expression of vitamin D3 receptor and retinoid receptors in human breast cancer: identification of potential heterodimeric receptors Int J Oncol 2004 25 1183 1191 15375571

[B32] Barhoover MA Hall JM Greenlee WF Thomas RS Aryl hydrocarbon receptor regulates cell cycle progression in human breast cancer cells via a functional interaction with cyclin-dependent kinase 4 Mol Pharmacol 2010 77 195 201 10.1124/mol.109.059675 19917880

[B33] Safe S Krishnan V Cellular and molecular biology of aryl hydrocarbon (Ah) receptor-mediated gene expression Arch Toxicol Suppl 1995 17 99 115 10.1007/978-3-642-79451-3_8 7786196

[B34] Darro F Cahen P Vianna A Decaestecker C Nogaret JM Leblond B Chaboteaux C Ramos C Pétein M Budel V Schoofs A Pourrias B Kiss R Growth inhibition of human in vitro and mouse in vitro and in vivo mammary tumor models by retinoids in comparison with tamoxifen and the RU-486 anti-progestagen Breast Cancer Res Treat 1998 51 39 55 10.1023/A:1006098124087 9877028

[B35] Paroni G Fratelli M Gardini G Bassano M Flora JM Zanetti A Guarnaccia V Ubezio P Centritto F Terao E Garattini A Synergistic antitumor activity of lapatinib and retinoids on a novel subtype of breast cancer with coamplification of ERBB2 and RARA Oncogene 2011 doi: 10.1038/onc.2011.506 22056878

[B36] Wakasugi T Izumi H Uchiumi T Suzuki H Arao T Nishio K Kohno K ZNF143 interacts with p73 and is involved in cisplatin resistance through the transcriptional regulation of DNA repair genes Oncogene 2007 26 5194 5203 10.1038/sj.onc.1210326 17297437

[B37] Tan K Shaw AL Madsen B Jensen K Taylor-Papadimitriou J Freemont PS Human PLU-1 Has transcriptional repression properties and interacts with the developmental transcription factors BF-1 and PAX9 J Biol Chem 2003 278 20507 20513 10.1074/jbc.M301994200 12657635

[B38] Chano T Ikebuchi K Ochi Y Tameno H Tomita Y Jin Y Inaji H Ishitobi M Teramoto K Nishimura I Minami K Inoue H Isono T Saitoh M Shimada T Hisa Y Okabe H RB1CC1 Activates RB1 Pathway and Inhibits Proliferation and Cologenic Survival in Human Cancer PLoS One 2010 5 e11404 10.1371/journal.pone.0011404 20614030 PMC2894861

[B39] Melkoumian ZK Peng X Gan B Wu X Guan J Mechanism of Cell Cycle Regulation by FIP200 in Human Breast Cancer Cells Cancer Res 2005 65 6676 6684 10.1158/0008-5472.CAN-04-4142 16061648

[B40] Patel AS Karagas MR Spencer SK Perry AE Nelson HH Gene-drug interaction at the glucocorticoid receptor increases risk of squamous cell skin cancer AACR Meeting Abstracts 2006 2006 1071 10.1038/sj.jid.5700798 17392827

[B41] Kim HH Sierke SL Koland JG Epidermal growth factor-dependent association of phosphatidylinositol 3-kinase with the erbB3 gene product J Biol Chem 1994 269 24747 24755 7929151

[B42] Paik J Kollipara R Chu G Ji H Xiao Y Ding Z Miao L Tothova Z Horner JW Carrasco DR Jiang S Gilliland DG Chin L Wong WH Castrillon DH DePinho RA FoxOs Are Lineage-Restricted Redundant Tumor Suppressors and Regulate Endothelial Cell Homeostasis Cell 2007 128 309 323 10.1016/j.cell.2006.12.029 17254969 PMC1855089

[B43] D'Alessio A De Luca A Maiello MR Lamura L Rachiglio AM Napolitano M Gallo M Normanno N Effects of the combined blockade of EGFR and ErbB-2 on signal transduction and regulation of cell cycle regulatory proteins in breast cancer cells Breast Cancer Res Treat 2010 123 387 396 10.1007/s10549-009-0649-x 19946741

[B44] LaBonte MJ Wilson PM Fazzone W Russell J Louie SG El-Khoueiry A Lenz H Ladner RD The Dual EGFR/HER2 Inhibitor Lapatinib Synergistically Enhances the Antitumor Activity of the Histone Deacetylase Inhibitor Panobinostat in Colorectal Cancer Models Cancer Res 2011 71 3635 3648 10.1158/0008-5472.CAN-10-2430 21464044 PMC3118510

[B45] Lui VWY Lau CPY Ho K Ng MHL Cheng SH Tsao S Tsang CM Lei KIK Chan AT Mok TSK Anti-invasion, anti-proliferation and anoikis-sensitization activities of lapatinib in nasopharyngeal carcinoma cells Invest New Drugs 2011 29 1241 1252 10.1007/s10637-010-9470-y 20571878

[B46] Schmidt M Fernandez de Mattos S van der Horst A Klompmaker R Kops GJPL Lam EW Burgering BMT Medema RH Cell Cycle Inhibition by FoxO Forkhead Transcription Factors Involves Downregulation of Cyclin D Mol Cell Biol 2002 22 7842 7852 10.1128/MCB.22.22.7842-7852.2002 12391153 PMC134724

[B47] Zhang Y Hamburger AW Heregulin regulates the ability of the ErbB3-binding protein Ebp1 to bind E2F promoter elements and repress E2F-mediated transcription J Biol Chem 2004 279 26126 26133 10.1074/jbc.M314305200 15073182

[B48] Ayroldi E Zollo O Bastianelli A Marchetti C Agostini M Di Virgilio R Riccardi C GILZ mediates the antiproliferative activity of glucocorticoids by negative regulation of Ras signaling J Clin Invest 2007 117 1605 1615 10.1172/JCI30724 17492054 PMC1865030

[B49] Takayama S Rogatsky I Schwarcz LE Darimont BD The glucocorticoid receptor represses cyclin D1 by targeting the Tcf-beta-catenin complex J Biol Chem 2006 281 17856 17863 10.1074/jbc.M602290200 16644723

[B50] Chen Y Dokmanovic M Stein WD Ardecky RJ Roninson IB Agonist and Antagonist of Retinoic Acid Receptors Cause Similar Changes in Gene Expression and Induce Senescence-like Growth Arrest in MCF-7 Breast Carcinoma Cells Cancer Res 2006 66 8749 8761 10.1158/0008-5472.CAN-06-0581 16951191

[B51] Hu MC Lee D Xia W Golfman LS Ou-Yang F Yang J Zou Y Bao S Hanada N Saso H Kobayashi R Hung M IkappaB kinase promotes tumorigenesis through inhibition of forkhead FOXO3a Cell 2004 117 225 237 10.1016/S0092-8674(04)00302-2 15084260

[B52] Liu B Ordonez-Ercan D Fan Z Edgerton SM Yang X Thor AD Downregulation of erbB3 abrogates erbB2-mediated tamoxifen resistance in breast cancer cells Int J Cancer 2007 120 1874 1882 10.1002/ijc.22423 17266042

