# Supplementary figures and images for: Gene expression changes as markers of early lapatinib response in a panel of breast cancer cell lines
Source: Mol Cancer. 2012 Jun 18;11:41. doi: 10.1186/1476-4598-11-41 (PMC3439312; doi:10.1186/1476-4598-11-41)

## Slide 1
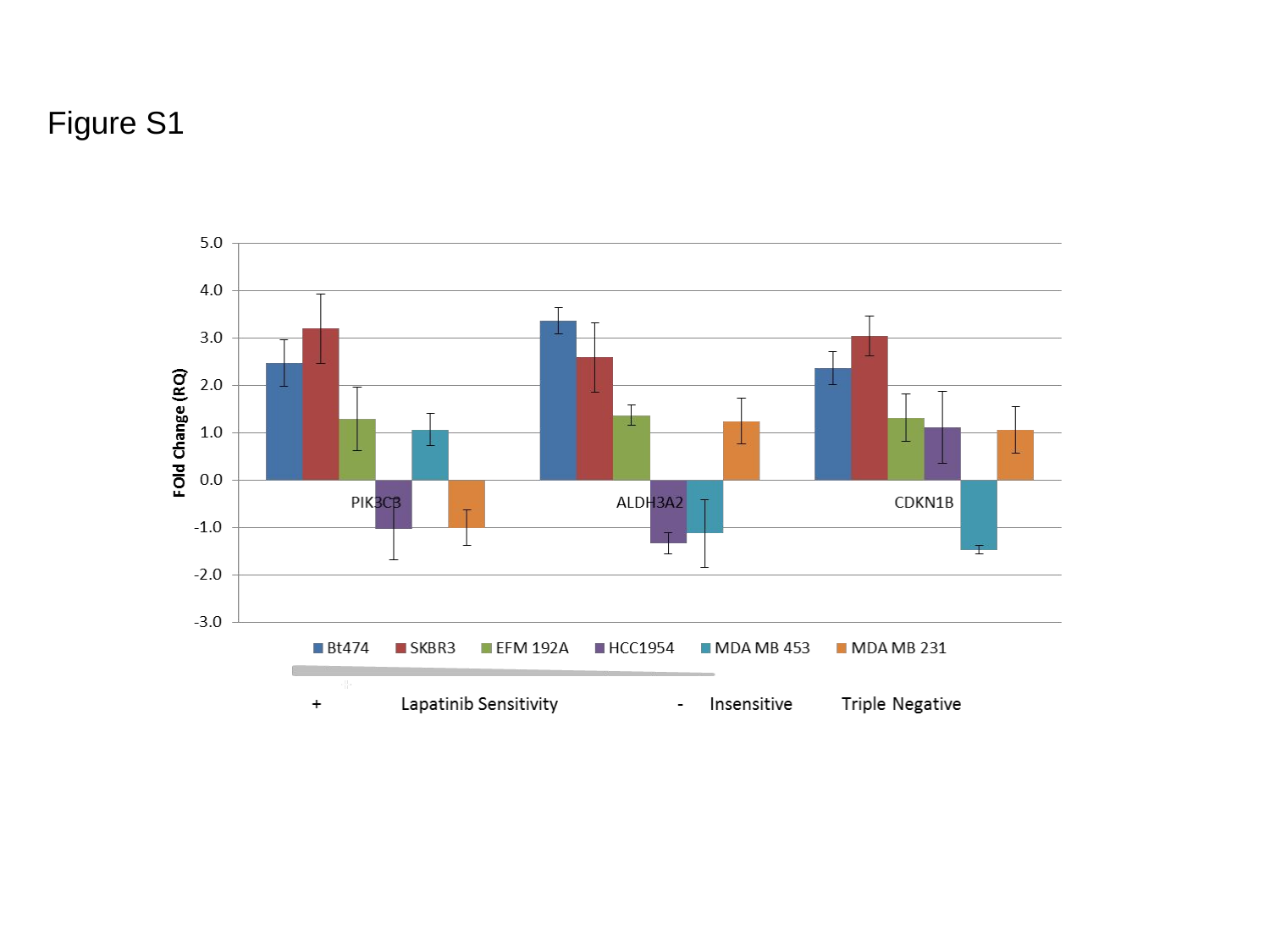

Figure S1

## Slide 2
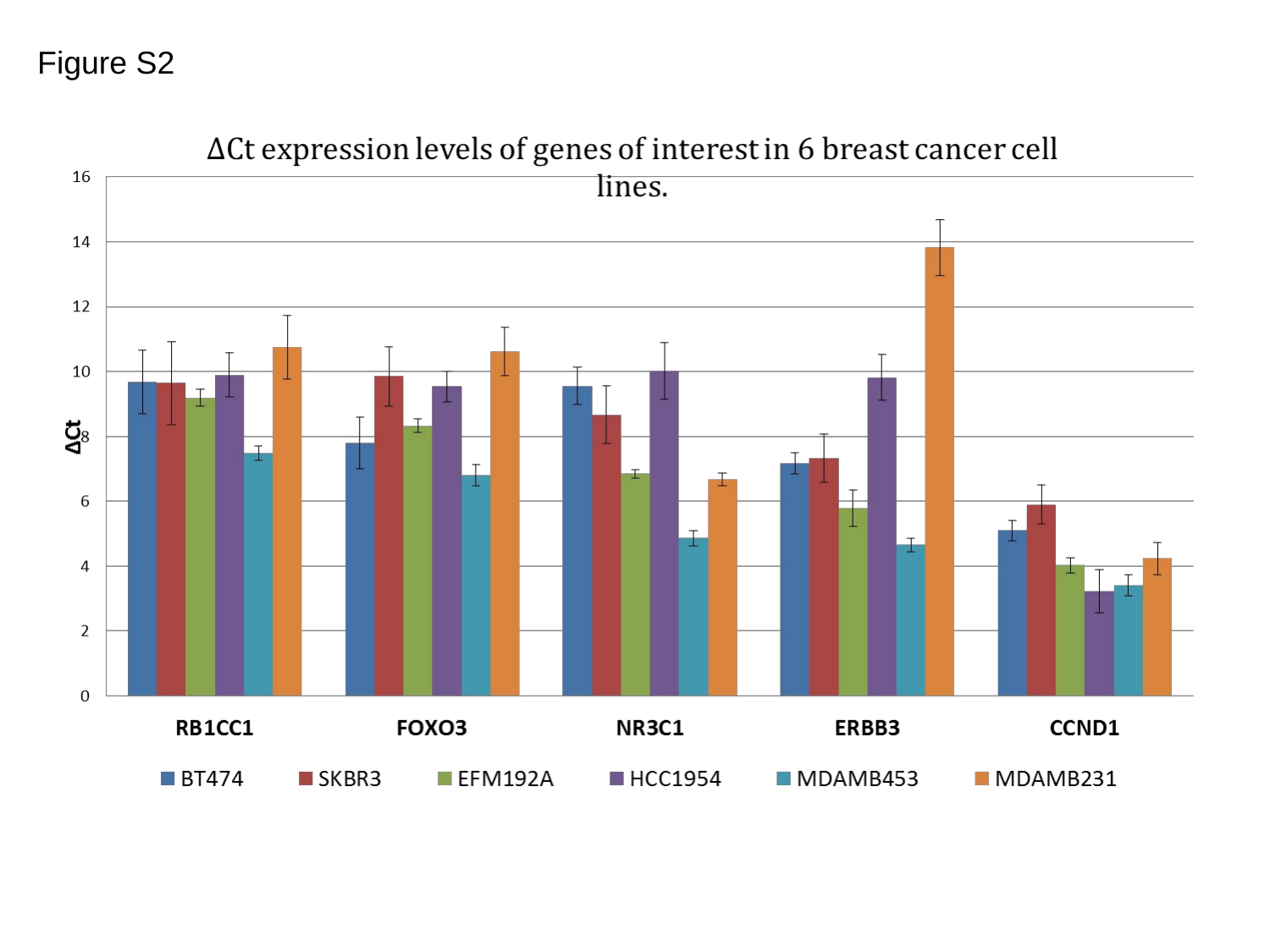

Figure S2

Supplement: Additional File 6 — Figure S1. Expression of PIK3C3, ALDH3A2 and CDKN1B across the six cell lines. Figure S2. Basal gene expression (ΔCt) of RB1CC1, FOXO3A, NR3C1, ERBB3 and CCND1 across the six cell lines. [file 1476-4598-11-41-S6.ppt]
